# Comparison of emergence agitation between succinylcholine and rocuronium-sugammadex in adults following closed reduction of a nasal bone fracture: a prospective randomized controlled trial

**DOI:** 10.1186/s12871-019-0907-3

**Published:** 2019-12-16

**Authors:** Seok-Jin Lee, Tae-Yun Sung, Choon-Kyu Cho

**Affiliations:** Department of Anaesthesiology and Pain Medicine, Konyang University Hospital, Myunggok Medical Research Center, Konyang University College of Medicine, 158, Gwangeodong-ro, Seo-gu, Daejeon, 35365 South Korea

**Keywords:** Emergence agitation, Rocuronium, Succinylcholine, Sugammadex

## Abstract

**Background:**

Sugammadex allows rapid recovery from rocuronium-induced neuromuscular blockade. Succinylcholine is often used for brief surgeries but is associated with myalgia, headache, histamine release, and increased lactate levels. Thus, we hypothesized that succinylcholine may affect emergence agitation (EA) and compared the effects of succinylcholine and rocuronium-sugammadex on EA in patients undergoing closed reduction of a nasal bone fracture under general anesthesia.

**Methods:**

Forty-two patients were prospectively enrolled and allocated randomly to the succinylcholine group (group SC) or the rocuronium-sugammadex group (group RS; each *n* = 21). Neuromuscular block and its reversal were achieved with succinylcholine and normal saline in group SC, whereas rocuronium and sugammadex were administered in group RS. After surgery, the incidence of EA as a primary outcome, the incidence of dangerous EA, and duration of EA as secondary outcomes were compared.

**Results:**

The incidence of EA was higher in group SC than in group RS (90.5% vs. 47.6%, respectively; relative risk [RR] 4.3; 95% confidence interval [CI] 1.2 to 15.7; *P* = .006). The incidence of dangerous EA increased in group SC compared to group RS (33.3% vs. 4.8%, respectively; RR 2.1; 95% CI 1.3 to 3.4; *P* = .045). The duration of agitation was longer in group SC than in group RS [106.5 (65.1) vs. 40.4 (26.0) sec; mean difference 66.1 s; 95% CI 31.0 to 101.1; effect size 1.3; *P* = .001).

**Conclusion:**

Succinylcholine increases the incidence, severity, and duration of EA compared to rocuronium-sugammadex in patients undergoing closed reduction of a nasal bone fracture.

**Trial registration:**

CRiS Registration number KCT0002673. Initial registration date was 31 January 2018 (Retrospectively registered)*.*

## Background

Despite a short surgical duration, general anesthesia is preferred for closed reduction of a nasal bone fracture due to extreme pain during the reduction manipulation, aspiration concerns, and better patient satisfaction with general anesthesia compared to local anesthesia [1]. However, closed reduction of a nasal bone fracture under general anesthesia is commonly associated with emergence agitation (EA) [2, 3].

Succinylcholine is a depolarizing neuromuscular blocking agent with a rapid onset time and a short duration of action; thus, it has been used for rapid sequence induction or short-duration procedures, such as closed reduction of a nasal bone fracture, despite its numerous side effects [2–5]. However, because sugammadex rapidly and effectively reverses any depth of steroidal neuromuscular blocking agent-induced neuromuscular blockade [6], the necessity for succinylcholine is controversial [7].

A case report suggested that rocuronium-sugammadex reduces the severity of agitation following electroconvulsive therapy by decreasing the increase in plasma lactate level compared to succinylcholine [8]. Additionally, succinylcholine can result in myalgia, headache, histamine release, increased intraocular pressure, and activation of the electroencephalogram [5, 9].

We hypothesized that succinylcholine would negatively affect EA and that rocuronium-sugammadex would reduce the incidence of EA compared to succinylcholine. Therefore, we designed this study to compare the effects of succinylcholine and rocuronium-sugammadex on EA in adult patients undergoing closed reduction of a nasal bone fracture.

## Methods

We conducted this randomized prospective double-blind study after receiving approval from the Institutional Review Board of Konyang University Hospital, Daejeon, Korea (October 2017; KYUH 2017–07–020-001) and obtained written informed consent from all participants. The study was registered with the Korean Clinical Research Information Service (https://cris.nih.go.kr, permit number: KCT0002673) and adhered to CONSORT checklist. Subjects included patients aged 20–65 years with American Society of Anesthesiologists physical status classification I–II who underwent general anesthesia for closed reduction of a nasal bone fracture. We excluded patients who were contraindicated to any of the study drugs (e.g., hyperkalemia, renal failure [glomerular filtration rate < 30 mL/min], hepatic dysfunction, severe burn or trauma, neuromuscular disease, personal history or family history of malignant hyperthermia), history of an allergic reaction to any of the study drugs, body mass index ≥30 kg/m^2^, presence of unstable vital signs, and any other fracture or injury requiring a combined operation in addition to reduction of a nasal bone fracture. Patients were allocated randomly (allocation ratio 1:1) to one of two groups (succinylcholine group: group SC or the rocuronium + sugammadex group; group RS) using a random number table generated using online randomization software (www.randomizer.org).

All patients fasted at least 8 h and received no premedication before the induction of anesthesia. Patients were blinded to their group allocation. All patients were monitored in the operating room using routine monitoring, including non-invasive automated blood pressure, pulse oximetry, electrocardiography, bispectral index (BIS; BIS VISTA™ monitor; Aspect Medical Systems, Norwood, MA, USA), and neuromuscular train-of-four (TOF) by acceleromyography (TOF-Watch SX®; Organon Ltd., Dublin, Ireland) on the adductor pollicis muscle. Preoxygenation was conducted with tidal volume breathing for 2 min using 8 L/min of 100% oxygen, and then anesthesia was induced with 2 mg/kg propofol and 1 μg /kg fentanyl. Succinylcholine (1 mg/kg) in group SC or rocuronium (0.6 mg/kg) in group RS was administered as a neuromuscular blocking agent to facilitate endotracheal intubation. After loss of consciousness and before administering the succinylcholine or rocuronium, the acceleromyograph was calibrated automatically in CAL-2 mode, and TOF stimulation was then initiated. Intubation was performed after confirming the disappearance of fasciculation or complete depression of the first twitch (T1) of the TOF in group SC, and after confirming a TOF count of zero in group RS. After intubation, volume-controlled mechanical ventilation was initiated at a tidal volume of 8 ml/kg and a respiratory rate of 12 breaths/min; then, the respiratory rate was adjusted to maintain an end-tidal carbon dioxide concentration of 30–40 mmHg. Anesthesia was maintained with 1.5–5 vol% of end-tidal concentration of sevoflurane and 50% nitrous oxide (N_2_O) to maintain the BIS at 40–60. Before the end of surgery, 0.3 mg ramosetron was injected intravenously to prevent postoperative nausea and vomiting in all patients. All events during the maintenance of anesthesia, such as patient-ventilator dyssynchrony (PVD), were recorded. PVD was denoted by significant changes in the volume, pressure and flow graphic waveforms of the ventilator [10].

At the end of the surgery, intranasal packing and a nasal splint were applied and 2 or 4 mg/kg sugammadex (Bridion®; MSD, Seoul, Korea) plus normal saline (total volume = 5 mL) was intravenously injected to reverse the neuromuscular blockade based on neuromuscular monitoring in group RS, whereas 5 mL normal saline was injected in group SC.

The study drugs were prepared and anesthesia was induced by an anesthesiologist who knew the patient allocation but was not involved in data collection. Data collection in the operating room was performed by another anesthesiologist who entered the operating room immediately after induction of anesthesia and was blinded to the patient allocations. The TOF monitor was covered with a surgical towel and exposure was only permitted to the anesthesiologist who prepared the study drugs. Preparation of the study drugs was masked to the data-collecting anesthesiologist using an opaque partition between the two anesthesiologists.

The same extubation criteria were applied to the groups; BIS ≥80, tidal volume ≥ 5 ml/kg, and respiratory rate 10–25 rate/min during spontaneous breathing, except TOF ratio ≥ 0.9 in group RS. Duration of anesthesia and surgery were defined as the time from induction of anesthesia to extubation and the time from packing intranasal Bosmin®-soaked gauze (1 mg/mL epinephrine solution; Je Il Pharm, Seoul, Korea) to complete the nasal splint after applying intranasal packing with saline-soaked Merocel® (polyvinyl acetate sponge, Medtronic Xomed, Jacksonville, FL, USA).

Emergence was defined as the time interval from discontinuation of the inhalational anesthetic to 5 min after extubation. The Ricker Sedation-Agitation Scale (RSAS) was used to assess EA [11], and the maximum score was recorded: 1 = unarousable, 2 = very sedated, 3 = sedated, 4 = calm and cooperative, 5 = agitated and calm to verbal instruction, 6 = very agitated, requiring restraint; 7 = pulling at the tracheal tube, trying to remove catheters, or striking the staff. RSAS ≥5 and = 7 were defined as EA and dangerous EA, respectively. The duration of any EA was measured. During emergence, time to first spontaneous respiration, time to first awakening response, such as eye opening to a verbal command or grimacing, and time to extubation were measured and recorded.

Postoperative pain and the sense of suffocation were recorded in the post-anesthetic care unit (PACU) on a 0–10 numeric rating scale (NRS, 0 = no sense of pain/suffocation, 10 = worst sense of pain/suffocation imaginable). In case of a NRS for pain > 4, 0.5 mg/kg fentanyl was injected intravenously and the patient was re-evaluated 10 min later. If a patient complained of nausea or vomiting, 10 mg metoclopramide was injected intravenously. Any complications, such as bitter taste, dizziness, headache, shivering, or respiratory depression, were also evaluated and recorded by the data-collecting anesthesiologist.

The primary outcome was incidence of EA (RSAS ≥5). The secondary outcomes were incidence of dangerous EA (RSAS = 7) and duration of EA. Other outcomes were frequency of PVD, time to first spontaneous respiration, time to first awakening response, time to extubation, NRS for postoperative pain and sense of suffocation, requirement for analgesics and/or antiemetics, and adverse events.

### Statistical analysis

In a pilot study, the incidence of EA was 92.0% in group SC (*n* = 12). A sample size of 19 patients per group was required to detect a 50% reduction in the incidence of EA with a power of 0.8 and a two-sided α-value of 0.05. Thus, we enrolled 21 patients per group considering potential dropout. The statistical analysis was conducted using SPSS software (ver. 18.0 for Windows; SPSS Inc., Chicago, IL, USA). The distribution of the numerical data was assessed with the Kolmogorov–Smirnov test. Continuous variables are presented as mean (standard deviation) or median and interquartile range, and were analyzed using Student’s *t*-test or the Mann–Whitney *U*-test where appropriate. Categorical variables were expressed as numbers (%) or numbers and analyzed by the χ^2^ test or Fisher’s exact test as appropriate. A *P*-value < .05 was considered significant.

## Results

**Fig. 1:**
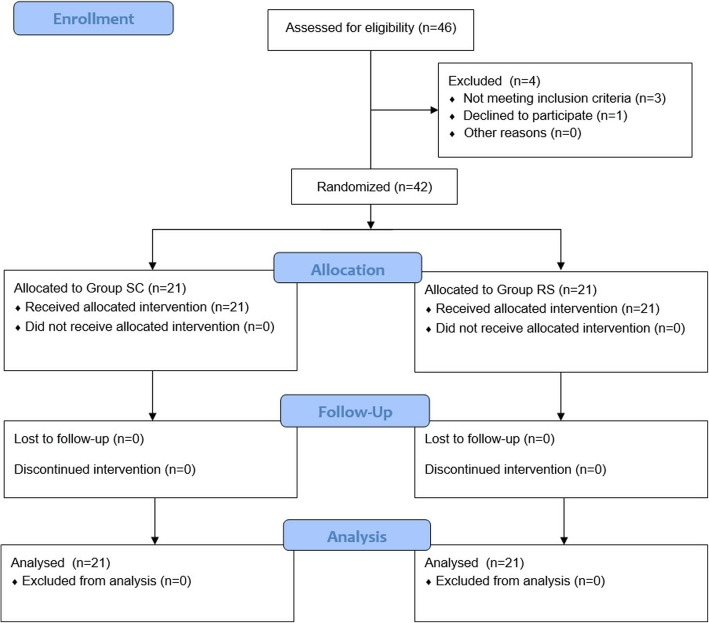
Flow chart. Group SC: succinylcholine group; Group RS: rocuronium + sugammadex group

In total, 46 patients were assessed for eligibility (28 November 2017 to 17 April 2018), and 4 were excluded; 3 patients had another fracture in addition to the nasal bone fracture and 1 patient refused to participate in the study. Consequently, 42 patients were randomly allocated to group SC or RS and analyzed (Fig. 1).

The demographic data were comparable between the two groups (Table 1).

**Table 1:** Demographic data and surgical details

	Group SC (*n* = 21)	Group RS (*n* = 21)
Age, yr	35.3 (14.5)	36.2 (14.2)
Gender (male), *n* (%)	14 (66.7)	14 (66.7)
Height, cm	169.8 (4.8)	167.7 (7.3)
Weight, kg	71.8 (11.0)	66.7 (5.6)
ASA (I/II), *n*	11/10	12/9
Intraoperative fluids, ml	100 [100–150]	150 [100–150]
Duration of surgery, min	17.6 (4.8)	16.5 (3.5)
Duration of anesthesia, min	32.4 (5.1)	32.3 (4.5)
Sugammadex (dose), *n* (%)		
2 mg/kg	NA	9 (42.9)
4 mg/kg	NA	12 (57.1)

Values are mean (standard deviation), median [IQR; interquartile range], numbers (%), or numbers

*Group SC* succinylcholine group, *Group RS* rocuronium + sugammadex group, *AS* American Society of Anesthesiologists physical status classification, *NA* not applicable

The incidence of EA was significantly higher in group SC than group RS (90.5% [19/21] vs. 47.6% [10/21], respectively; relative risk [RR] 4.3; 95% CI 1.2 to 15.7; *P* = .006; Table 2). The incidence of dangerous EA was also significantly higher in group SC than group RS (33.3% [7/21] vs. 4.8% [1/21], respectively; RR 2.1; 95% CI 1.3 to 3.4; *P* = .045; Table 2). The duration of agitation was significantly more prolonged in group SC than group RS [106.5 (65.1) sec vs. 40.4 (26.0) sec, respectively; mean difference 66.1 s; 95% CI 31.0 to 101.1; effect size 1.3; *P* = .001; Table 2). During surgery, PVD was more frequent in group SC than group RS (23.8% [5/21] vs. 0% [0/21], respectively; RR 2.3; 95% confidence interval [CI] 1.6 to 3.3; *P* = .048; Table 2). Time to spontaneous respiration, time to first awakening response, and time to extubation did not differ between the groups (Table 2). In the PACU, the NRS for pain, the NRS for sense of suffocation, and the requirement for analgesics and/or an antiemetic drug did not differ between the groups (Table 2).

**Table 2:** Intraoperative and recovery data

	Group SC (*n* = 21)	Group RS (*n* = 21)	Mean difference or RR (95% CI)	*P*
In operating room				
Patient-ventilator dyssynchrony, *n* (%)	5 (23.8)	0 (0)	2.3 (1.6 to 3.3)	0.04
Emergence agitation, *n* (%)	19 (90.5)	10 (47.6)	4.3 (1.2 to 15.7)	0.01
Dangerous emergence agitation, *n* (%)	7 (33.3)	1 (4.8)	2.1 (1.3 to 3.4)	0.04
Duration of agitation, sec	106.5 (65.1)	40.4 (26.0)	66.1 (31.0 to 101.1)	0.001
Time to spontaneous respiration, min	5.9 (2.1)	5.6 (2.0)	0.3 (−0.9 to 1.6)	0.60
Time to first awakening response, min	6.9 (1.7)	7.1 (1.7)	−0.3 (−1.4 to 0.7)	0.54
Time to extubation, min	8.3 (1.7)	7.4 (1.7)	0.9 (−0.1 to1.9)	0.09
In PACU				
NRS for pain	2.8 (2.4)	3.5 (2.4)	−0.7 (−2.2 to 0.7)	0.34
NRS for sense of suffocation	7.1 (2.0)	6.5 (2.6)	0.6 (−0.8 to 2.1)	0.39
Fentanyl, *n* (%)	3 (14.3)	5 (23.8)	NA	0.70
Metoclopramide, *n* (%)	0	1 (4.8)	NA	> 0.99

Values are mean (standard deviation), numbers (%) or numbers

*Group SC* succinylcholine group, *Group RS* rocuronium + sugammadex group, *RR* relative risk, *CI* confidence interval, *NRS* numerical rating scale (0 = no sense of pain/suffocation, 10 = worst sense of pain/suffocation imaginable), *NA* not applicable, *PACU* post-anesthesia care unit

The rate of adverse events was also similar between the groups (Table 3).

**Table 3:** Adverse events

	Group SC (*n* = 21)	Group RS (*n* = 21)	*P*
Headache	2 (9.5)	5 (23.8)	0.41
Dizziness	2 (9.5)	0 (0)	0.48
Bitter taste	2 (9.5)	0 (0)	0.48
Myalgia	1 (4.8)	0 (0)	> 0.99
Dry mouth	1 (4.8)	0 (0)	> 0.99
Shivering	0 (0)	1 (4.8)	> 0.99
Nausea	0 (0)	1 (4.8)	> 0.99
Vomiting	0 (0)	0 (0)	NA

Values are numbers (%)

*Group SC* succinylcholine group, *Group RS* rocuronium + sugammadex group, *NA* not applicable

## Discussion

This study demonstrated that neuromuscular blockade using succinylcholine and spontaneous recovery of neuromuscular function is associated with more frequent, dangerous, and longer EA compared to that encountered after administering rocuronium-induced neuromuscular blockade with reversal by sugammadex in adult patients undergoing closed reduction of a nasal bone fracture.

Several studies have investigated the prevention of EA by comparing inhalation anesthetics and intravenous anesthetics or by using analgesics or sedatives [3, 12–16]; potent analgesics (remifentanil, fentanyl, nefopam), *N*-methyl-D-aspartate receptor antagonists (ketamine, magnesium sulfate, and tramadol), α2-aderenoreceptor agonists (clonidine and dexmedetomidine), and propofol have a protective effect on EA, while inhalation anesthetics with low blood/gas partition coefficients (sevoflurane and desflurane), doxapram, and benzodiazepine premedication increase the risk of EA.

To our knowledge, this is the first randomized controlled study to compare the effects of different kinds of neuromuscular blocking agents with reversal on EA after general anesthesia. Our results suggest that different types of neuromuscular blocking agents (depolarizing vs. nondepolarizing) and/or reversal (spontaneously vs. by sugammadex) may affect the characteristics of EA.

EA is more common following closed reduction of a nasal bone fracture than following other surgeries [2, 3, 15]. We speculate that the use of sevoflurane as an inhalation anesthetic agent with low blood-gas solubility is a causative factor in the relatively high incidence of EA after this surgery. Closed reduction of a nasal bone fracture is a short-duration surgery, so inhalation anesthetics permitting rapid recovery after surgery have been favored [2, 3]. However, inhalation anesthesia has an increased incidence of EA compared with total intravenous anesthesia [15, 17]. Additionally, sevoflurane anesthesia causes increases in brain lactate and glucose concentrations, and elevated brain lactate and glucose concentrations are positively correlated with the EA score [18]. Another factor that may increase EA is intranasal packing because packing can lead to an abrupt sense of suffocation when a patient’s consciousness is restored. A previous study [17] on adults undergoing nasal surgery found that intranasal packing following nasal surgery was not a risk factor for EA; the authors explained that nasal obstruction by nasal packing had little influence on their patients’ breathing because patients who undergo nasal surgery are chronic mouth breathers due to their nasal disease. However, that study did not include patients undergoing closed reduction of a nasal bone fracture [17]. Most patients who require closed reduction of a nasal bone fracture are not chronic mouth breathers because their fracture resulted from acute trauma [1]. This is supported by the NRS scores in the PACU of our patients: although previous studies [15, 17, 19] have demonstrated that pain is one of the most important risk factors for EA in adults, scores for suffocation were substantially higher than pain scores in both study groups (Table 2).

In the present study, succinylcholine had a more negative association with EA in terms of incidence, severity, and duration, compared to rocuronium-sugammadex. Although the precise mechanism of EA is unclear, possible explanations are as follows. First, EA may be related to metabolic changes because the use of succinylcholine increases lactate and potassium concentrations compared to rocuronium-sugammadex [8, 20]. An increase in brain lactate level could indicate greater neuronal activity and is linked with a tendency to manifest EA [18]. Increased potassium level may affect potassium channels in the lateral nucleus of the amygdala, which is associated with regulating stress-induced behavior [21]. Second, although the succinylcholine and rocuronium doses used in this study correspond to the typical doses recommended to facilitate intubation, the different depth of neuromuscular block between the groups may have affected EA. The duration of a complete succinylcholine block (dose of 1 mg/kg) in a patient with normal plasma cholinesterase is 3–7 min and the duration of action for rocuronium (dose of 0.6 mg/kg) is 35–45 min [5, 9]. In our study, the difference in duration of action between the two neuromuscular blocking agents resulted in more frequent PVD in group SC than in group RS (23.8% vs. 0%, respectively). Rocuronium provided deep or moderate depth of neuromuscular block in all patients in group RS until the end of surgery on TOF monitoring. Insufficient neuromuscular block in group SC may increase the administration of sevoflurane to suppress PVD. Moreover, although we adjusted the concentration of sevoflurane according to an equal BIS of 40–60 in both groups, the shallow depth of the neuromuscular block in group SC increased electromyographic (EMG) activity, which can result in an increased concentration of sevoflurane to decrease the BIS, regardless of the actual level of sedation [22]. However, different concentrations of sevoflurane and resulting depths of anesthesia did not affect the EA [23]. Third, succinylcholine can increase intraocular and intragastric pressure, cause flushing due to histamine release, and have undesirable autonomic effects [9]. It is not known exactly how these factors affect the occurrence of EA, but it is likely that they had a negative effect on EA.

In the present study, the incidence of EA in group SC was higher than in a previous study of closed reduction of nasal bone fracture, despite the use of the same neuromuscular blocking agent (succinylcholine) and the same inhalation anesthetics (sevoflurane-N_2_O) [3]. This difference might be related to the use of different EA assessment parameters. In the previous study [3], the authors reported an EA incidence of 45.0% using Aono’s scale [24] and EA was defined when the Aono score was ≥3. In other words, they did not include cases as EA when the Aono score was 2. Aono’s scale defines 2 as “not calm, but could be easily calmed”, which is very similar to a RSAS of 5 (= agitated and calm to verbal instruction), which we used to consider EA in our study. If an Aono’s score of 2 were to be considered EA, the incidence of EA would be 95.0%, which is comparable to the 90.5% in our study.

The use of rocuronium-sugammadex reduces the frequency of headache and myalgia following electroconvulsive therapy compared to succinylcholine [4]. However, adverse events were comparable between the two groups in our study. The reason for the discordance in the results of adverse events may be mainly attributed to the different interventions (electroconvulsive therapy vs. closed reduction of a nasal bone fracture); it might also be related to the fact that our study was not fully powered to detect differences in adverse events.

Although rocuronium-sugammadex has several advantages over succinylcholine (e.g., predictable reversal of any depth of rocuronium-induced neuromuscular block, reduced incidence of PVD during mechanical ventilation, reduced incidence and severity of EA, etc.), sugammadex is significantly more expensive. A systemic review of rocuronium-sugammadex and succinylcholine failed to produce an estimate cost-effectiveness due to a lack of underlying relevant clinical data [25]. Conclusive pharmacoeconomic assessment of these drugs may require further clinical studies.

This study had several limitations. First, objective neuromuscular monitoring was not included in the extubation criteria of group SC because tetanic fade does not occur at a clinically appropriate concentration of succinylcholine [26]. Although clinically significant residual neuromuscular block was not observed in this study, there was the possibility of residual neuromuscular block in some patients who received succinylcholine because succinylcholine has high interpatient variability in duration of action (range 1.3–44 min) [27]. Incomplete recovery of neuromuscular function may be partly attributed to the high incidence of EA in group SC by causing distress and agitation during emergence. Second, the blood concentration of carbon dioxide was not measured in this study. A decrease in cerebral blood flow due to hypocarbia, and acidosis due to hypercarbia, may alter consciousness and contribute to the occurrence of EA [28]. However, in this study, the end-tidal carbon dioxide was adjusted from 30 to 40 mmHg in both groups; therefore, the effect of blood carbon dioxide on EA would be similar in both groups. Finally, it is unclear whether the results of this study are due to an EA-preventive effect of rocuronium-sugammadex or an EA-inducing effect of succinylcholine, or both. However, considering that rocuronium or sugammadex intrinsically has no analgesic or sedative effects that could contribute to reduce the incidence of EA, the results of this study may be mainly due to the EA-inducing effect of succinylcholine. On the other hand, even if rocuronium-sugammadex has intrinsic efficacy in preventing EA, it is also uncertain which of the two mainly contributed to reducing the incidence of EA. Further studies using rocuronium with different kinds of reversal agents (e.g., antiacetylcholinesterase vs. sugammadex vs. spontaneous recovery) or different kinds of neuromuscular blocking agents (e.g., rocuronium vs. vecuronium) with reversal by sugammadex are needed.

## Conclusion

Rocuronium-induced neuromuscular block and recovery using sugammadex are effective for decreasing the incidence, severity, and duration of EA compared to succinylcholine following closed reduction of a nasal bone fracture.

## Data Availability

The datasets analyzed during the current study are available from the corresponding author on reasonable request.

## Abbreviations

EA: Emergence agitation
NA: Not applicable
NRS: Numerical rating scale
PACU: Post-anesthesia care unit
RS: Rocuronium-sugammadex
SC: Succinylcholine

## References

[1] Al-Moraissi EA, Ellis E (2015). Local versus general anesthesia for the management of nasal bone fractures: a systematic review and meta-analysis. J Oral Maxillofac Surg.

[2] Lee YS, Baek CW, Kim DR, Kang H, Choi GJ, Park YH (2016). Comparison of hemodynamic response to tracheal intubation and postoperative pain in patients undergoing closed reduction of nasal bone fracture under general anesthesia: a randomized controlled trial comparing fentanyl and oxycodone. BMC Anesthesiol.

[3] Kim YS, Chae YK, Choi YS, Min JH, Ahn SW, Yoon JW (2012). Comparative study of emergence agitation between sevoflurane and propofol anesthesia in adults after closed reduction of nasal bone fracture. Korean J Anesthesiol.

[4] Saricicek V, Sahin L, Bulbul F, Ucar S, Sahin M (2014). Does rocuronium-sugammadex reduce myalgia and headache after electroconvulsive therapy in patients with major depression?. J ECT.

[5] Lee C (2009). Goodbye suxamethonium!. Anaesthesia..

[6] Keating GM (2016). Sugammadex: a review of neuromuscular blockade reversal. Drugs..

[7] Caldwell JE, Miller RD (2009). Clinical implications of sugammadex. Anaesthesia.

[8] Postaci A, Tiryaki C, Sacan O, Ornek D, Kalyoncu M, Dikmen B (2013). Rocuronium-sugammadex decreases the severity of post-electroconvulsive therapy agitation. J ECT.

[9] Orebaugh SL (1999). Succinylcholine: adverse effects and alternatives in emergency medicine. Am J Emerg Med.

[10] Mellott KG, Grap MJ, Munro CL, Sessler CN, Wetzel PA (2009). Patient-ventilator dyssynchrony: clinical significance and implications for practice. Crit Care Nurse.

[11] Riker RR, Picard JT, Fraser GL (1999). Prospective evaluation of the sedation-agitation scale for adult critically ill patients. Crit Care Med.

[12] Polat R, Peker K, Baran I, Bumin AG, Gülöksüz TC, Dönmez A (2015). Comparison between dexmedetomidine and remifentanil infusion in emergence agitation during recovery after nasal surgery: a randomized double-blind trial. Anaesthesist.

[13] Jee YS, You HJ, Sung TY, Cho CK (2017). Effects of nefopam on emergence agitation after general anesthesia for nasal surgery: a prospective, randomized, and controlled trial. Medicine.

[14] Lee SJ, Choi SJ, In CB, Sung TY (2019). Effects of tramadol on emergence agitation after general anesthesia for nasal surgery: a retrospective cohort study. Medicine.

[15] Yu D, Chai W, Sun X, Yao L (2010). Emergence agitation in adults: risk factors in 2,000 patients. Can J Anesth.

[16] Dahmani S, Stany I, Brasher C, Lejeune C, Bruneau B, Wood C (2010). Pharmacological prevention of sevoflurane and desflurane-related emergence agitation in children: a meta-analysis of published studies. Br J Anaesth.

[17] Kim HJ, Kim DK, Kim HY, Kim JK, Choi SW (2015). Risk factors of emergence agitation in adults undergoing general anesthesia for nasal surgery. Clin Exp Otorhinolaryngol.

[18] Jacob Z, Li H, Makaryus R, Zhang S, Reinsel R (2012). Metabolomic profiling of children’s brains undergoing general anesthesia with sevoflurane and propofol. Anesthesiology.

[19] Rim JC, Kim JA, Hong JI, Park SY, Lee JH, Chung CJ (2016). Risk factors of emergence agitation after general anesthesia in adult patients. Anesth Pain Med.

[20] Sabo D, Jahr J, Pavlin J, Philip B, Shimode N, Rowe E (2014). The increases in potassium concentrations are greater with succinylcholine than with rocuronium-sugammadex in outpatient surgery: a randomized, multicentre trial. Can J Anaesth.

[21] McLott J, Jurecic J, Hemphill L, Dunn KS (2013). Development of an amygdalocentric neurocircuitry-reactive aggression theoretical model of emergence delirium in posttraumatic stress disorder: an integrative literature review. AANA J.

[22] Dahaba AA (2005). Different conditions that could result in the bispectral index indicating an incorrect hypnotic state. Anesth Analg.

[23] Frederick HJ, Wofford K, de Lisle DG, Schulman SRA (2016). Randomized controlled trial to determine the effect of depth of anesthesia on emergence agitation in children. Anesth Analg.

[24] Aono J, Ueda W, Mamiya K, Takimoto E, Manabe M (1997). Greater incidence of delirium during recovery from sevoflurane anesthesia in preschool boys. Anesthesiology..

[25] Chambers D, Paulden M, Paton F, Heirs M, Duffy S, Hunter JM (2010). Sugammadex for reversal of neuromuscular block after rapid sequence intubation: a systematic review and economic assessment. Br J Anaesth.

[26] Jonsson M, Dabrowski M, Gurley DA, Larsson O, Johnson EC (2006). Activation and inhibition of human muscular and neuronal nicotinic acetylcholine receptors by succinylcholine. Anesthesiology..

[27] Dell-Kuster S, Levano S, Burkhart CS, Lelais F, Zemp A, Schobinger E (2015). Predictors of the variability in neuromuscular block duration following succinylcholine: a prospective, observational study. Eur J Anaesthesiol.

[28] Viswanath O, Kerner B, Jean YK, Soto R, Rosen G (2015). Emergence delirium: a narrative review. J Anesthesiol Clin Sci.

